# BioNAR: an integrated biological network analysis package in bioconductor

**DOI:** 10.1093/bioadv/vbad137

**Published:** 2023-09-29

**Authors:** Colin McLean, Anatoly Sorokin, Thomas Ian Simpson, James Douglas Armstrong, Oksana Sorokina

**Affiliations:** Edinburgh Cancer Research Centre, Institute for Genetics and Cancer, University of Edinburgh, Edinburgh EH4 2XU, United Kingdom; Biological Systems Unit, Okinawa Institute of Science and Technology, Onna, Okinawa 904-0495, Japan; School of Informatics, University of Edinburgh, Edinburgh EH8 9AB, United Kingdom; School of Informatics, University of Edinburgh, Edinburgh EH8 9AB, United Kingdom; C omputational Biomedicine Institute (IAS-5/INM-9), Forschungszentrum Jülich, Jülich, Germany; School of Informatics, University of Edinburgh, Edinburgh EH8 9AB, United Kingdom

## Abstract

**Motivation:**

Biological function in protein complexes emerges from more than just the sum of their parts: molecules interact in a range of different sub-complexes and transfer signals/information around internal pathways. Modern proteomic techniques are excellent at producing a parts-list for such complexes, but more detailed analysis demands a network approach linking the molecules together and analysing the emergent architectural properties. Methods developed for the analysis of networks in social sciences have proven very useful for splitting biological networks into communities leading to the discovery of sub-complexes enriched with molecules associated with specific diseases or molecular functions that are not apparent from the constituent components alone.

**Results:**

Here, we present the Bioconductor package BioNAR, which supports step-by-step analysis of biological/biomedical networks with the aim of quantifying and ranking each of the network’s vertices based on network topology and clustering. Examples demonstrate that while BioNAR is not restricted to proteomic networks, it can predict a protein’s impact within multiple complexes, and enables estimation of the co-occurrence of metadata, i.e. diseases and functions across the network, identifying the clusters whose components are likely to share common function and mechanisms.

**Availability and implementation:**

The package is available from Bioconductor release 3.17: https://bioconductor.org/packages/release/bioc/html/BioNAR.html.

## 1 Introduction

Biotechnology has made rapid advances in recent years with massive steps forward in both the sensitivity and throughput of methods to analyse biological samples across multiple levels. Arguably, the best-known examples are based on data from high-throughput DNA and RNA sequencing, but there are an increasing number of reports using proteomics, metabolomics, and connectomics data. Much of our understanding of the biological processes that underpin our health and well-being is limited to isolated analyses of small sets of components. Integration of these into network models can facilitate identification of key functional interactions, pathways, and complexes that are required for normal function and that, when perturbed, lead to disease.

Communication, or information transfer, between the components of a network is a common feature across biological scales. Therefore, it is not entirely surprising that methods designed to analyse information flow or communication in social networks have proven to be well suited for the analysis of biological networks in general and proteomic networks in particular (Ma and Zeng 2003, Wunderlich and Mirny 2006, Li *et al.* 2011).

Proteomic data are typically represented via static undirected protein–protein interaction (PPI) networks, where vertices represent the proteins obtained from mass-spectrometry experiments and edges represent the structural protein interactions connecting them. From a PPI network one can extract many statistical measures of the network’s topology and its fundamental properties and use these to gain insight and make predictions about the underlying data (Vidal *et al.* 2011, Bánky *et al.* 2013). For example, ‘scale-free’ (Barabási and Albert 1999) properties and small world paths found in many biological networks are widely used to identify ‘hub’ molecules, which often encode disease related proteins (Vidal *et al.* 2011).

Protein networks can be large (1000 s of proteins) and contain the components of multiple known signalling pathways all joined together by other proteins whose role in the network is poorly understood. Therefore, it is useful to divide PPI networks into communities (or clusters) based on their connecting architecture, under the assumption that shared network topology (interconnectedness) may correlate with shared function (or dysfunction) (Zhu *et al.* 2007, Fernandez *et al.* 2009, Klemmer *et al.* 2009). To obtain this, functional and disease over-representation of clusters can be calculated to reveal the clusters that are significantly enriched for specific annotations. This kind of analysis, e.g. was used to help link together the molecular pathways in the synapse, which underpin healthy neuronal function, as well as synaptic diseases (Pocklington *et al.* 2006a).

Many stand-alone software tools have been developed to address the basic steps required for the analytical steps described above. For example, Cytoscape (Shannon *et al.* 2003) supports interactive reconstruction and visual representation of molecular networks, clustering, and estimation of the main centrality measures. Various other tools exist for functional enrichment analysis based on the GO, KEGG (Kanehisa and Goto 2000), and Reactome (Gillespie *et al.* 2022) ontologies (Maere *et al.* 2005, Subramanian *et al.* 2005, Eden *et al.* 2009, Sherman *et al.* 2022). The *igraph* package in R supports building a network, estimating centrality measures and several types of clustering (Gabor and Tamas 2006).

What is missing is a platform that enables a robust pipeline approach to be deployed at scale. To achieve this within the Bioconductor environment, we developed BioNAR. It provides a topologically based network analysis pipeline, enabling users to load networks generated and/or annotated using their lab’s own metadata, thus making the tool as widely applicable and flexible as possible. The pipeline approach facilitates iterative analytical designs and stochastic sampling for enhanced statistical robustness.

BioNAR supports a range of network analysis functions and integrates with several existing R packages to maximize its utility (Supplementary Table S1). Full list of dependencies can be found on the Bioconductor page (https://bioconductor.org/packages/3.16/bioc/html/BioNAR.html). Importantly, BioNAR fills key methodological gaps to allow for the interrogation of biomedical networks from functional and disease perspectives. We provide methods for identification of ‘bridging’ proteins—those that can participate in multiple communities simultaneously and play an important role in signal propagation across the network (Nepusz *et al.* 2008). Another important feature, not implemented elsewhere, is the estimation of the topological overlap for different annotations. It was recently demonstrated that within a large-scale molecular network, the location of each sub-network (module) of disease associated genes correlates with its pathobiological relationship to other disease sub-networks, e.g. diseases with overlapping modules showed significant similarities at the level of gene co-expression patterns, clinical phenotype, and comorbidity (Menche *et al.* 2015). Conversely, diseases residing in separated network neighbourhoods appear to be more phenotypically distinct. We implemented overlap/separation estimation in BioNAR, making it applicable not only for diseases, but for any annotation pair of interest.

As our previous and ongoing work is related to the synaptic proteome, we illustrate the package functionality with two publicly available synaptic networks (first and second case studies). However, BioNAR is not limited to synaptic or even proteomic networks and can be applied to any biological network, e.g. patient network, gene–disease (third case study), or disease–disease interactions with any customized annotation.

## 2 Results

### 2.1 Implementation

**Figure 1.**
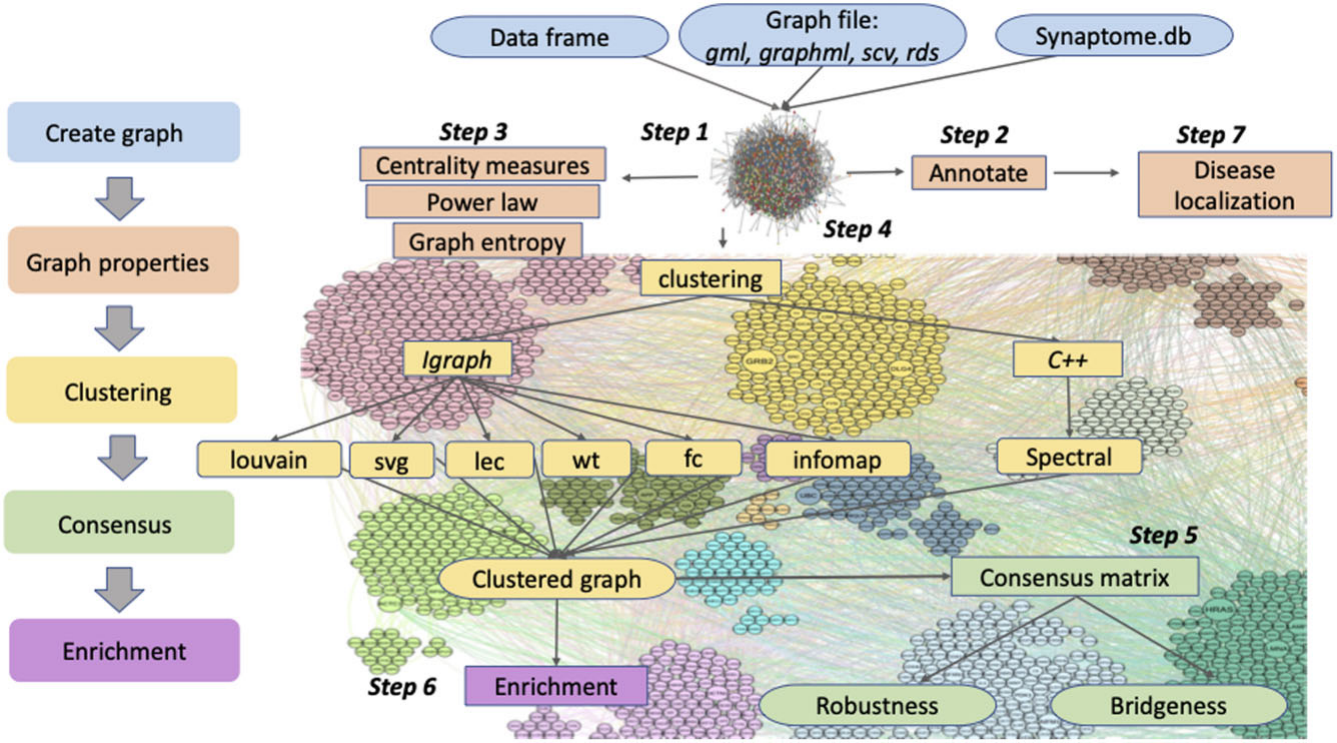
Network analysis pipeline implemented in BioNAR package. Although the process is often interactive, the general flow starts with graph creation and proceeds as illustrated on the left. Each of the steps indicated corresponds to the analysis steps described in Section 2. Colours correspond to the respective processing stages (left).

We developed the Bioconductor package, BioNAR, to support the analysis of biological networks based around a high-level pipeline presented in Fig. 1. We describe each of these key functions below (also see the package vignette here: https://www.bioconductor.org/packages/devel/bioc/vignettes/BioNAR/inst/doc/BioNAR_overview.html).

#### 2.1.1 Step 1. Creating a network instance

BioNAR implements networks as R data frames, where each row corresponds to a vertex interactor pair and where each vertex has a unique vertex_ID. Alternatively, a network can be imported from standard graph file formats including gml, using the igraph’s package built-in functionality. BioNAR also allows network import for specific synaptic protein set/synaptic compartments/brain region directly from the *Synaptome.db* package (Sorokina *et al.* 2022). An example of this is shown for the presynaptic case study 2. For constructing the protein interaction networks described here, we used the NCBI Gene Entrez ID as a unique *vertex_ID* for each node/protein.

#### 2.1.2 Step 2. Adding annotation to the vertices

Once a network is constructed, vertices are typically annotated with categorical or continuous metadata. Annotations are handled in a three-column data frame format, where the first column contains the annotation term ID, the second the annotation term name, and the third column the *vertex_*ID (Entrez ID in our case). All annotation terms for the same *vertex_ID* are collected and stored as a semicolon-separated string in the vertex annotation. BioNAR is designed to assign the results of any vertex calculation as a new vertex attribute, which allows intermediate results to be stored directly within the network. This supports reproducibility as many algorithms used in network analysis have a stochastic component, so each invocation can create a range of slightly different results.

BioNAR supports automatic annotation of the proteomic networks with NCBI gene names, GO annotations, and gene–disease association values, but can also be used for any annotation data the user would be interested in, such as gene expression values, pathway membership data and so on. Examples of adding custom annotation are presented in Case 2. For further analysis, annotation strings are converted into semicolon-separated lists to be stored as vertex attributes.

#### 2.1.3 Step 3. Estimating network vertex properties and underlaying structure

The BioNAR package directly supports calculation of the following network vertex centrality measures, many of which are implemented in igraph (Gabor and Tamas 2006): degree (DEG), betweenness (BET), clustering coefficient (CC), semi-local centrality (SL), mean shortest path (mnSP), page rank (PR), and standard deviation of the shortest path (sdSP) (see *igraph* manual for details). Vertex centrality values can be added as vertex attributes (*calcCentrality*) or returned as an R matrix (*makeCentralityMatrix*), depending on user preference. Any other numerical characteristics, calculated for vertices and represented in a matrix form, can also be stored as a vertex attribute (*applyMatrixToGraph*).

To enable comparison of an observed network’s vertex centrality values to those of an equivalently sized randomized graph, we enabled three randomization models including G(n, p) Erdos–Renyi model (Erdős and Rényi 1959), Barabási–Albert model (Barabási and Albert 1999), and the derivation of a new randomized graph from a given graph by iteratively and randomly adding/removing edges. To examine a network for underlying structure (i.e. not a random network), one can test a network’s degree distribution for evidence of scale-free structure and compare it to an equivalent randomized network model. For this, we used the R *PoweRlaw* package (version 0.50.0) (Gillespie 2015), which uses a goodness-of-fit approach to estimate the lower bound and the scaling parameter of the discrete power-law distribution for the optimal description of the graph degree distribution.

For proteomic networks where we also have multi-condition gene expression data, scale-free structure can also be tested by using the expression data to perform a perturbation analysis on the network to measure network entropy (Teschendorff *et al.* 2015), which corresponds to a degree of randomness in the local pattern information flux around single genes. This kind of analysis is most useful for comparing a control relative to a perturbed network (e.g. wild-type versus cancer, untreated versus treated), where vertices with low entropy rate appear to be the most important players in disease propagation. However, for the assessment of scale-free structure, we do not actually require gene expression data as it is based solely on the network topology. BioNAR follows the procedure described in Teschendorff *et al.* (2015): all vertexes are artificially assigned a uniform weight then sequentially perturbed with the global entropy rate (SR) after each protein’s perturbation being calculated and plotted against the log of the protein’s degree. In the case of scale-free or approximate scale-free topology, a bi-modal, bi-phasic behaviour is observed (Teschendorff *et al.* 2015) (see Case 2).

#### 2.1.4 Step 4. Clustering

BioNAR supports a non-exhaustive set of commonly used clustering algorithms. These are modularity-maximization-based algorithms, including the popular agglomerative ‘Fast-Greedy Community’ algorithm (fc) (Clauset *et al.* 2004), process driven agglomerative random walk algorithm ‘Walktrap’ (wt) (Pons and Latapy 2006), and coupled Potts/Simulated Annealing algorithm ‘SpinGlass’ (sg) (Reichardt and Bornholdt 2006, Traag and Bruggeman 2009), the divisive spectral-based ‘Leading-Eigenvector’ (lec) (Newman 2006) and fine-tuning (Spectral) (McLean *et al.* 2016) algorithms, and the hierarchical agglomerative ‘Louvain’ algorithm (louvain) (Blondel *et al.* 2008). We also include a non-modularity information-theory-based algorithm ‘InfoMAP’ (infomap) (Rosvall and Bergstrom 2008, Rosvall *et al.* 2009).

All algorithm implementations, apart from Spectral, were imported from R’s igraph package (Gabor and Tamas 2006). The Spectral algorithm (McLean *et al.* 2016) was written in C++ and wrapped in R within a satellite CRAN package *rSpectral* (https://cran.r-project.org/web/packages/rSpectral/index.html), linked to BioNAR (more details in Supplementary Methods).

Depending on the purpose of the study all clustering algorithms can be applied to the network under investigation simultaneously, with each algorithm’s community membership stored as a vertex attribute. The user also has the option to select specific clustering algorithms to run over their network, since running all clustering algorithms over the large network is likely to be resource intensive.

A common phenomenon when applying Modularity-based clustering algorithms over networks of a large size, is to end with large, or ‘super’, communities which masks network substructure. In this situation, we provide the user the facility to re-cluster these large/super communities in a hierarchical manner, applying the same, or potentially a differing, clustering algorithm at each iteration (using the BioNAR *recluster* function).

To compare the usefulness of different clustering algorithms on a network, a summary matrix can be created, consisting of: the maximum Modularity obtained (mod), the number of detected communities (*C*), the number of singlet communities (Cn1), the number of communities with size ≥100 (Cn100), the fraction of edges lying between communities (mu), the size of the smallest community (Min. *C*) and the largest community (Max. *C*), the average (Mean *C*), median (Median *C*), first quartile (first Qu. *C*), and third quartile (third Qu. *C*) of community size (Table 1).

**Table 1.** Clustering summary for the MASC network.^a^

	*N*	mod	*C*	Cn1	Cn100	Mu	Min. *C*	First Qu. *C*	Median *C*	Mean *C*	3rd Qu. *C*	Max. *C*
RWMod	101	0.4419	13	0	0	0.2927	2	3	4	7.769	8	29
lec	101	0.4393	8	0	0	0.3862	3	7.75	15	12.62	16.25	22
wt	101	0.3922	21	3	0	0.4187	1	2	3	4.81	6	25
fc	101	0.4842	8	0	0	0.3089	3	5	12.5	12.62	20	23
infomap	101	0.4753	13	0	0	0.3618	2	5	6	7.769	11	21
louvain	101	0.4698	7	0	0	0.3293	5	8.5	17	14.43	19.5	23
sgG1	101	0.4822	10	0	0	0.3333	2	4.25	8.5	10.1	14.75	21
sgG2	101	0.4424	15	0	0	0.4715	2	5.5	6	6.733	8.5	11
sgG5	101	0.3217	29	2	0	0.6382	1	2	3	3.483	4	8
spectral	101	0.4495	13	1	0	0.4268	1	4	7	7.769	10	15

a Shown are the maximum modularity obtained (mod), the number of detected communities (*C*), the number of singlet communities (Cn1), the number of communities with size ≥100 (Cn100), the fraction of edges lying between communities (mu), the size of the smallest community (Min. *C*) and the largest community (Max. *C*), the average (Mean *C*), median (Median *C*), first quartile (first Qu. *C*), and third quartile (third Qu. *C*) of community size. Three instances for SGg (1,2,5) in Table 1 correspond to one ‘SpinGlass’ (sg) in Section 2.1.4 and are designed to test the clustering with predefined number of clusters.

To test the robustness of communities found by a clustering algorithm, a consensus matrix is built by randomly selecting a proportion (by default 80%) of the network vertices and rerunning the clustering algorithm (by default set to 500 times). The functionality provided in the R package *clusterCons* (Simpson *et al.* 2010) then sums up the elements of the consensus matrix found in the same cluster and divides this by the total number of entries in the matrix, providing community robustness values in a range from zero, indicating low confidence in the community existing, to one, indicating high confidence in the cluster existing.

The BioNAR package provides functionality to visualize a network’s community structure with our implementation of cluster-driven layout, which is suitable for even the largest network (i.e. tens of thousands of vertices and millions of edges). This layout splits the network into clusters, lays out each cluster individually, and then combines individual layouts with the *igraph* function *merge_coords*, so that each distinct community is shown independently and painted in a unique colour.

To allow comparison of networks with different structures, we also implement a normalized modularity measure (Parter *et al.* 2007, Takemoto and Borjigin 2011, Takemoto 2012, 2013) (see package documentation for more detail https://bioconductor.org/packages/3.16/bioc/manuals/BioNAR/man/BioNAR.pdf).

#### 2.1.5 Step 5. Bridgeness and identifying ‘influential’ vertices

The clustering algorithms we used in Step 5 place each vertex into a single cluster, which in many cases is an oversimplification. In the context of proteomic networks, we know that proteins are often present in multiple copies, in multiple sub-complexes sometimes serving very different biological functions. The ‘bridgeness’ metric measures the probability that a vertex belongs to more than one community at the same time. Thus, estimating the bridgeness allows each protein in the network to be ranked according to its predicted importance for propagating signals through the network based on architecture alone.

Bridgeness can be estimated from the consensus matrix calculated in Step 4, taking values between 0—implying a vertex clearly belongs in a single community, and 1—implying a vertex forms a ‘global bridge’ across every community with the same strength (Nepusz *et al.* 2008, Nepusz *et al.* 2012).

Bridgeness becomes especially informative when combined with other vertex centrality measures, e.g. semi-local centrality, which considers the nearest and next to the nearest vertex neighbours. It also lies between 0 and 1 indicating whether the vertex is likely to have local influence.

Plotting bridgeness against semi-local centrality, allows us to categorize the local and global influence of each vertex within a network given only the network structure (see Case study 2). BioNAR supports the comparison of bridgeness against any vertex centrality measure (or any normalized numeric vertex value) of the user’s choice, e.g. against Page Rank.

#### 2.1.6 Step 6. Studying the overlap or separation of annotation pairs

Given two annotations that are distributed across a network, a common query is to find the points of intersection where the two annotation sets overlap (or segregate). To support such queries, we implemented the algorithm from Menche *et al.* (2015), which tests if the observed mean shortest paths between two distinct annotation sets, superimposed on a network, is significant compared to a randomly annotated network (see also Supplementary Methods).

This method is often applied to disease annotations although any similar type of annotation will work. The BioNAR command *calcDiseasePairs* calculates the observed overlap between two annotation sets on a network, and compares this to a single instance of the network with annotations randomly permuted; this is useful for a quick estimate of how likely the overlap is simply a random occurrence.

To calculate the significance of observed overlaps (or separations) the observed annotation pairs on the network the command *runPermDisease* should be used. This compares the overlap against multiple permutations of the network (where the user can define the number of permutations). Executing this command, which may take time depending on the number of permutations chosen, generates a results table containing the overlap of each annotation pair with *P*-value, *P* adjusted by Bonferroni test, and *q*-value.

#### 2.1.7 Step 7. Enrichment analysis

Over-representation analysis (ORA) is a common approach to identify annotation terms that are significantly over- or under-represented in a given set of vertices compared to a random distribution.

In biological networks GeneOntology terms and Pathway names are amongst the most frequently used. ORA differs from Gene Set Enrichment Analysis as the latter use numerical values associated with genes, such as expression value, while the former relies on null hypothesis tests, such as the hypergeometric test statistics. The most commonly used Gene Ontology analysis can be performed with dedicated Bioconductor tools, such as *clusterProfiler* (Wu *et al.* 2021). To keep the package as general purpose as possible and avoid ties to any annotation source, we used the Bioconductor package *fgsea* (Korotkevich *et al.* 2021) to implement ORA functionality on top of arbitrary string vertex annotation and vertex grouping, obtained e.g. by clustering. We represent the results of ORA as a R data frame, with rows representing the group of vertices and columns the *P*-value enrichment values for set of annotations terms under study. We also provide *P*-value, Benjamini–Hochberg-adjusted *P*-value, size of overlap, and list of vertices that contribute to the annotation term.

### 2.2 Case studies

The following three case studies illustrate how the BioNAR functionalities can be used for the networks of different size and origin.

#### 2.2.1 MASC network

**Figure 2.**
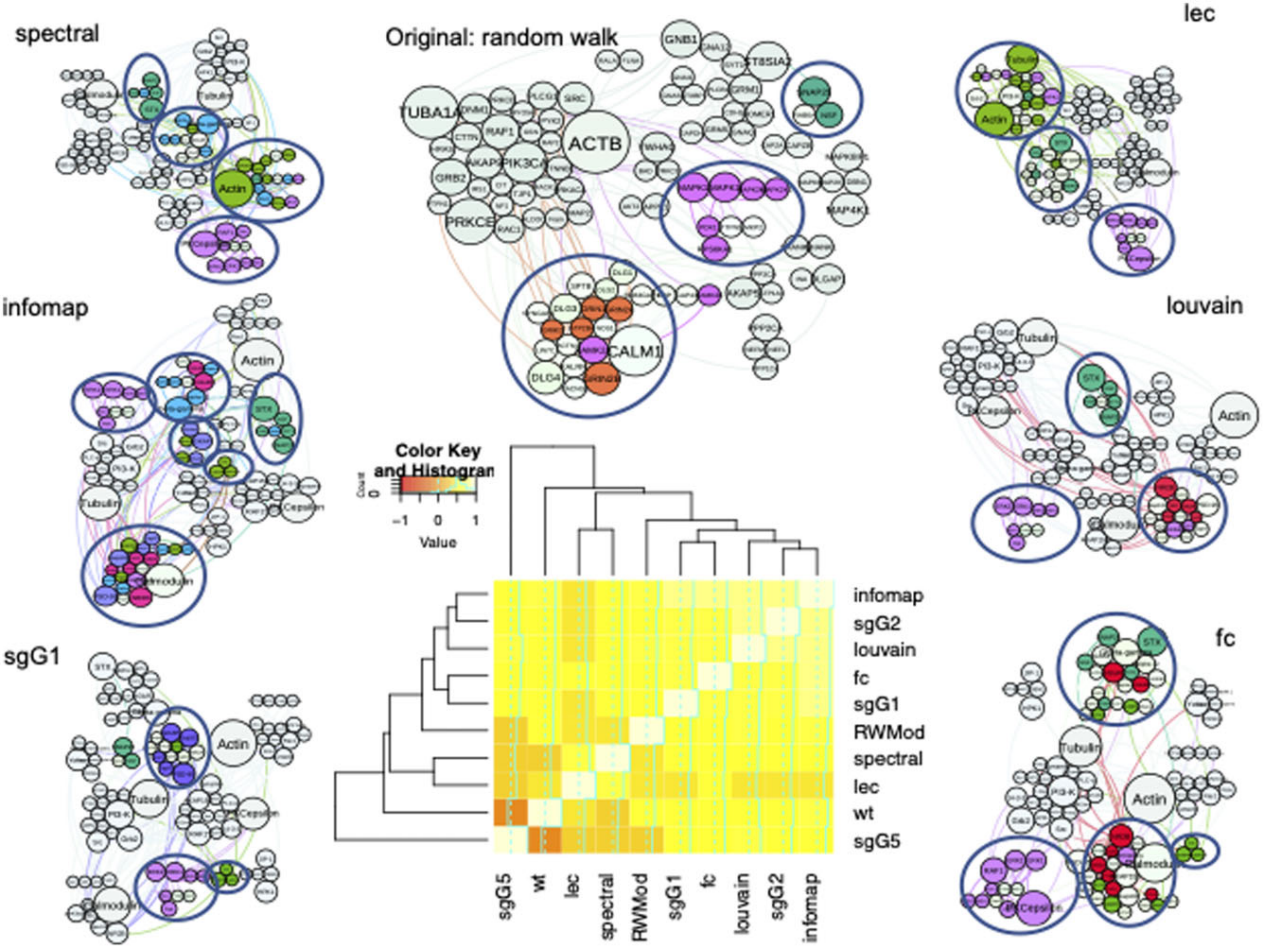
Clustering results for Case Study 1 (MASC network) for six algorithms. The colour code corresponds to the protein functional families described in Pocklington *et al.* (2006a) as follows: red—channels and receptors, light green—cell adhesion and cytoskeleton, dark green—synaptic vesicles/protein transport, light blue—G-proteins and modulators, purple—MAGUKs/adaptors/scaffolds, and maroon—kinases. Highlighted are only the clusters with significant enrichment (*P*-value <.05, p.adj).

The first test case chosen is a previously studied NMDA receptor complex known as the MASC network (Pocklington *et al.* 2006b). This is a relatively small PPI network, representing a protein complex surrounding the mammalian NMDA receptors and consists of 101 proteins with 246 interactions (Pocklington *et al.* 2006b). It is a good example of the type of network that would come from a typical pull-down experiment. The original MASC study provided an analysis of the organization and underlying functionality of the modularized MASC complex, and was clustered using the Random Walk algorithm (Newman and Girvan 2004) as shown in Table 1 (RWMod) and the top centre of Fig. 2. The analyses used in the original study predated most of the tools listed here and were achieved using almost entirely manually curated datasets and bespoke code developed for the specific study. Using BioNAR, we replicated the cluster analysis using our set of algorithms and visualized the results using Gephi (www.gephi.org). The clustering summary is presented in Table 1, which demonstrates that all algorithms give similar clustering structure with 8–13 communities, composed of an average of 5–10 proteins each.

We performed ORA to test if the obtained community structures (clusters) have meaningful enrichment for functional annotation terms, using the functional protein family annotations from the original paper (Pocklington *et al.* 2006b). As can be seen from Fig. 2, all clustering algorithms split the network into similar functional units. With all considered algorithms, we find two persistent clusters, containing proteins associated with: (i) synaptic vesicles/protein transport, containing 2–5 proteins of six possible family members, and (ii) kinases, containing 4–7 from 18 family proteins. In addition, the following families appear enriched in the clusters produced by the majority of algorithms, e.g. (i) channels and receptors, containing 5–6 proteins of eight family members (RWMod, Louvain, fc, infomap, and sgG2 algorithms), (ii) cell adhesion and cytoskeleton, containing 3–7 proteins from the respective family (spectral, lec, wt, fc, infomap, and sgG1), (iii) G-proteins and modulators, containing 3–5 from 17 possible proteins (spectral, infomap, sgG2, and sgG5), and (iv) MAGUKs/adaptors/scaffolds (3–5 from 12 family proteins, identified by wt, infomap, sgG1, and sgG2). The *P*-value and p.adj values for the respective algorithms can be found in Supplementary Table S2. In terms of over-representation, the highest level of significant enrichment is observed in the clustering produced by the infomap algorithm, which provides the split into six distinct significantly enriched functional communities (Fig. 2 and Supplementary Table S2).

We compared the performance of different clustering algorithms by estimating Newman’s Reduced Mutual Information index (Newman *et al.* 2020), which enables pairwise comparison of classifications of the same sets of objects (we used *clustAnalytics* package, https://CRAN.R-project.org/package=clustAnalytics*)*. Infomap, Louvain, fc, sgG1, and sgG2 algorithms split the network in similar way to the original clustering, while lec, spectral and, especially, wt give more divergent results (Fig. 2, bottom).

#### 2.2.2 Presynaptic network

A scale up, in both size and complexity, from the MASC network in Study 1 is the study of an entire subcellular compartment’s PPI network that integrates data from multiple experiments. For that we constructed a proteome network for the entire presynaptic compartment using the R package Synaptome.db (Sorokina *et al.* 2022): 2304 vertices were extracted from published studies of this compartment, which were combined with PPIs from *Synaptome.db* to obtain a Largest Connected Component containing 1780 vertices connected by 6620 edges.

**Figure 3.**
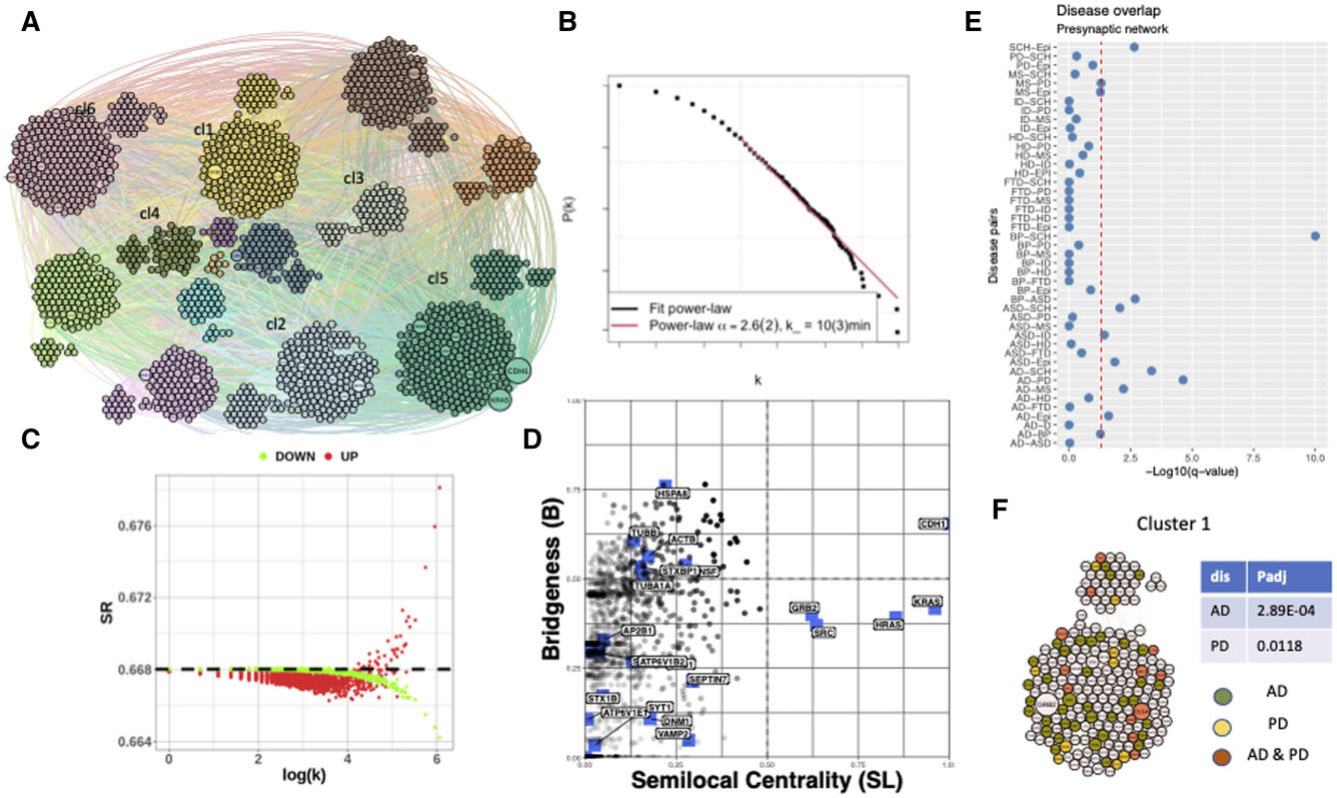
Presynaptic network, analysis results. (A) Clustering results from applying the Louvain algorithm, with clusters (cl) assigned a unique colour. (B) Power-law fit—shown the log–log plot of the CDF of presynaptic PPI network degree distribution [*P*(*k*)], versus its degree (*k*), with the best fitting power-law distribution to the network data highlighted in red. (C) Entropy plot for the presynaptic network. Each protein was perturbed through over-expression (red) and under-expression (green), with the global graph entropy rate (SR) after each protein perturbation being plotted against the log of the protein’s degree. (D) Bridgeness results shown for Louvain algorithm, highlighted are the genes most frequently found in presynaptic compartment. (E) Disease–disease overlap for presynaptic compartment, red dotted line shows the confidence cut-off (*q*-val <0.05). Abbreviations: Alzheimer disease (AD), Bipolar disorder (BP), Autistic spectral disorder (ASD), Epilepsy (Epi), Parkinson disease (PD), Schizophrenia (SCH), Frontotemporal dementia (FTD), Intellectual Disability (ID), Huntington disease (HD), Multiple sclerosis (MS). (F) Cluster 1 in details with highlighted proteins associated with AD and PD.

First, we examined the network for underlying structure by testing the network’s degree distribution against the randomized network model and found evidence of scale-free structure (alpha exponent to a power-law distribution of 2.6 was calculated using the PowerLaw function, Fig. 3B). Supporting evidence for scale-free structure was obtained by performing a perturbation analysis on the network (Fig. 3C). As in Teschendorff *et al.* (2015), proteins were set to initial values of 2 with perturbed values of 14 when modelling activity and set to initial values of 16 with perturbed values of 14 when modelling inactivity. We observed a bi-modal response between gene over-expression and degree, and opposing bi-phasic response relative to over/under-expression between global graph entropy rate and degree, typical for the scale-free topology.

To detect community structure in the presynaptic PPI network, we used a set of nine clustering algorithms implemented in BioNAR. A summary of the clustering results from each algorithm applied to the presynaptic network is presented in Supplementary Table S3. Each algorithm produces different communities. For illustration purposes, we selected clustering results produced using the ‘Louvain’ algorithm, which gives a reasonably small number of functionally enriched communities (14), one of the highest modularity values (0.469), no singletons, and communities distributed in size from 18 (the smallest) to 284 (the largest) vertices (Fig. 3A) for further analysis.

From a core set of five clustering algorithms including infomap, Spectral, sgG1, Louvain, and fc algorithms (Supplementary Table S4), we identified 324 (324/1780 ∼18%) potential bridging proteins (Bridgeness value ≥0.5) within the presynaptic PPI network; 15 of these were identified by all five clustering algorithms (15/323, 4%), 109 by three or more (109/323, 33%). Bridging proteins were found distributed through the entire network, and included STXBP1, ACTN1, CDH1, APP, VCP, PTPRF CAMK2A, and CAMK2B; many of these proteins are understood to be important in forming cytoplasmic scaffolds that organize and connect the synaptic vesicle with the presynaptic membrane or are involved in multiple signalling cascades (Chua *et al.* 2010).

We checked whether any bridging proteins were annotated with one or more of the more common synaptopathies, specifically: Alzheimer disease (AD), Bipolar disorder (BP/BD), Autism spectrum disorder (ASD), Epilepsy (Epi), Parkinson disease (PD), Schizophrenia (SCH), Frontotemporal dementia (FTD), and Intellectual disability (ID). Of the 324 proteins, 169 (169/324∼51%, *P* = 8.56E-05) were annotated with at least one disease. Of 15 proteins bridging proteins found in all five clustering algorithms, 10 (10/15 ∼67%) were found associated with at least one synaptic disease given in our set (Supplementary Table S4). The plot of bridgeness, using the Louvain clustering algorithm, against semi-Local centrality is shown in Fig. 3D. We highlight the 19 bridging proteins found most frequently in the presynaptic compartment [found in over 15 presynaptic studies (Sorokina *et al.* 2022)]. Among these, six have high bridgeness values, thus likely have a global influence over the network: HSPA8 (AD, PD, SCH), ACTB (PD, SCH), TUBB (FTD, SCH), STXBP1(AD, PD, FTD, SCH, ID), NSF (PD), and TUBA1A (SCH). Additionally, we highlighted CDH1, as it has both high values for bridgeness and semi-local centrality and is associated with ID. High local centrality values were also observed for GRB2, HRAS, and NRAS, which are recognized as local hubs participating in many signalling cascades (also known as ‘party hubs’).

Many neurological disorders are co-morbid and share similarities in clinical phenotype. To test whether common synaptic molecular mechanisms might underpin these disease similarities, we performed disease separation analysis for the disease annotation sets mentioned above (Fig. 3E, more detail in Supplementary Methods). The pair showing the most overlap was BD-SCH (q.val = 1.19E-09), followed by AD-PD (q.val = 1.64E-05), AD-SCH (q.val = 4.12E-04), and BP-ASD (q.val = 7.2E-04) (Supplementary Table S5), which are already known for their comorbidity. Distribution analysis of disease-associated proteins over the network combined with ORA showed that the majority of diseases, for instance, AD, PD, Epi, ASD, SCH, ID, and even BP are over-represented in Cluster 1.

To test whether specific synaptic functions were associated with clusters, we annotated our network with the SynGo (Koopmans *et al.* 2019) (release 20210225) and schizophrenia-annotations (SHAnno) (Lips *et al.* 2012), and performed ORA. The full cluster over-representation results are presented in Supplementary Table S6, but briefly, we found Cluster 1 over-represented with ‘structural constituent of post-synaptic density’ (p.adj = 5.18E-06), ‘regulation of post-synaptic neurotransmitter receptor activity’ (p.adj = 7.27E-03), and ‘excitability’ (p.adj = 7.64E-4) terms, which may indicate that Cluster 1 is enriched with membrane-associated proteins, the majority of which have been annotated elsewhere as being both pre- and post-synaptic. The co-occurrence of enrichment for specific synaptic functions and disease associations in the network clusters points to shared molecular mechanisms.

#### 2.2.3 Human disease network

In 2007, Barabasi and colleagues curated and published a Human ‘diseasome’ network along with an analysis of two of its natural projections: Human Disease Network (HDN) and Disease Gene Network (DGN) (Goh *et al.* 2007). The diseasome was built by collecting and annotating relationships between known human diseases, and disease-causing gene mutations. The diseasome is a bipartite network as it only contains the edges between two vertex types, i.e. ‘disease’ and ‘gene’ vertices. Projections of a bipartite network result in mono-type graphs, where a pair of vertices are connected if, and only if, that pair was connected to the same, but opposite, typed vertex in the original bipartite network. Therefore, the HDN graph contains disease vertices and connections between them if both diseases are linked by mutations in the same gene, while the DGN graph contains only gene vertices, which are connected only if both genes are associated with the same disease. We have reconstructed all three networks from the Supplementary Material in Goh *et al.* (2007) producing networks with largest connected components containing ∼46% (diseasome), 40% (HDN), and 50% (DGN) of all possible vertices, respectively.

**Figure 4.**
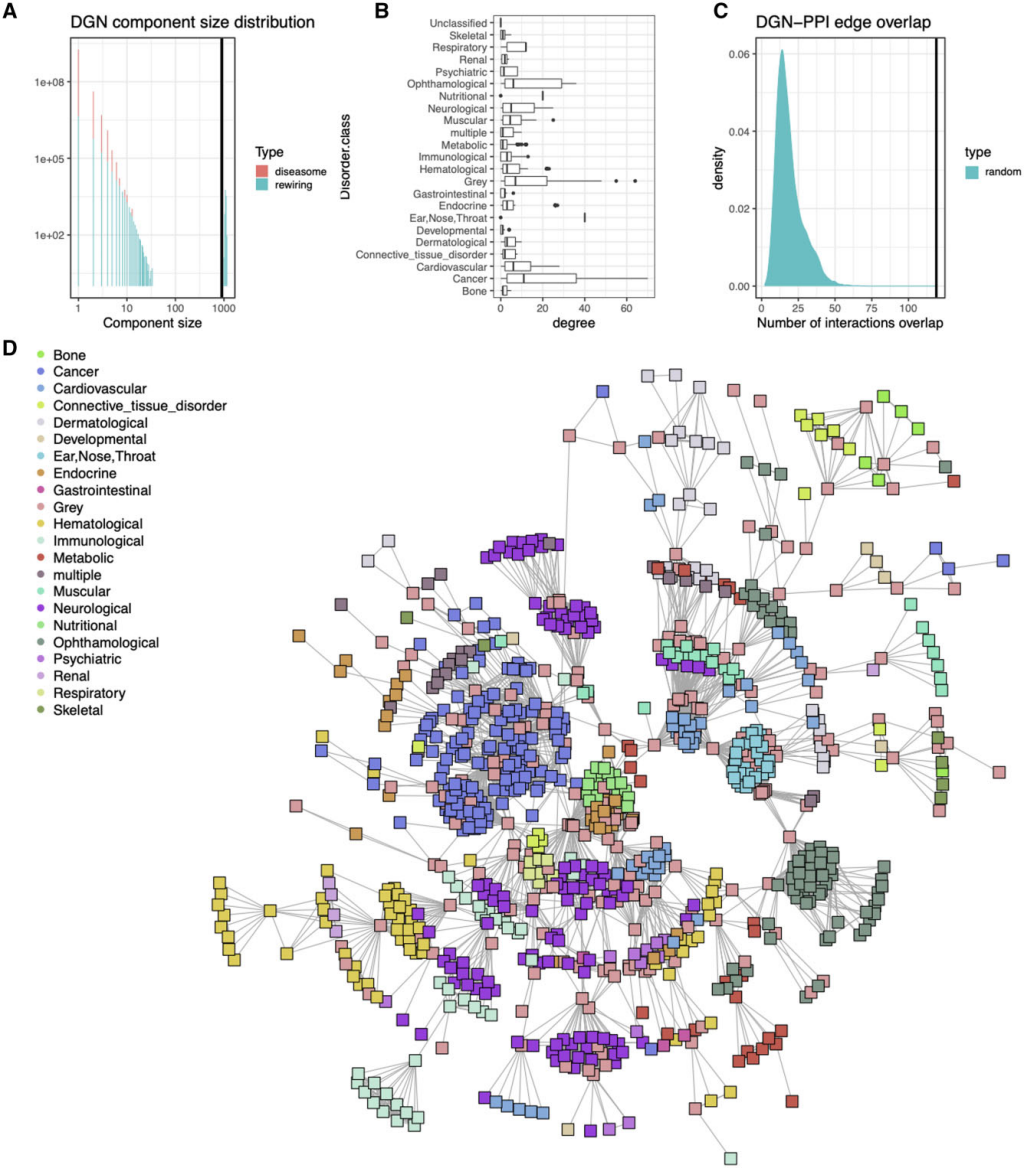
DGN analysis performed with BioNAR. (A) Distribution of connected component sizes in DGN. Red columns correspond to component sizes in the original network, while green to randomly perturbed networks. Vertical line on the graph corresponds to the size of the giant component of the original DGN it includes 50.8% of nodes. Like in the original Barabasi paper its size (903) is significantly lower (*P*-value <10–3) than the average size of the giant component (1088 ± 21) in the set of rewired networks. (B) Degree distribution of genes annotated with different disorder classes. It can be seen that high-degree genes come almost exclusively from the disorder class Cancer. A further disorder class ‘Grey’, which also contains high-degree genes, is associated with many disorders (Goh *et al.* 2007). (C) Number of observed interactions between genes annotated by the same disorder type (vertical line) and distribution of the expected numbers from randomized network. (D) The largest connected component of DGN. Nodes are coloured according to the disorder class.

When we randomly permuted connections in the original diseasome bipartite network and compared the size of the Largest Connected Component (LCC) in all three networks, we found each to be significantly smaller (*P*-value <.002) compared to that in the unperturbed networks (see Fig. 4A). It was discussed in Goh *et al.* (2007) that the small size of the LCC was probably caused by preferential attachment of genes within the same disorder class.

Here, we focussed on the analysis of DGN (Fig. 4D). Unlike the networks in the other case studies, analysed in this article, the DGN network does not show a scale-free distribution (Supplementary Fig. S1).

The degree distribution for the DGN network only follows a power-law at the extreme high-degree tail (Supplementary Fig. S1). This can be attributed to the DGN’s construction since we project an incomplete bipartite disease–gene network onto gene nodes. The low-degree genes in such a network result from our sparse knowledge about ‘less studied’ diseases, while the well-studied diseases, such as cancers, with high coverage in genes, lead to high degree and well-established connectivity of vertices (Fig. 4B), which reflects a power-law-like behaviour over this degree range.

Similar to Goh *et al.* (2007), we compared the inferred interactions between genes in the DGN to experimentally observed ones. While Goh *et al.* (2007) used a manually curated PPI network for the DGN, we extracted a PPI from published synaptic proteome data available via the synaptome.db Bioconductor package (Sorokina *et al.* 2022) by filtering out vertices from the ‘neuroPPI’ network, which were not found in the DGN. We calculated the number of disease module interactions as the number of PPI edges found between genes annotated to the disorder class given in DGN (Fig. 4C). We observed a smaller number of disease module interactions (119), than that found in Goh *et al.* (2007) (290) (which is not surprising given we are focussing on just synaptic interactions), but this was still significantly larger than expected by chance (19 ± 9.1) (Fig. 4C), indicating that the proteins encoded by genes associated with the same disease are highly likely to physically interact.

Thus, we reproduced a major part of the (Goh *et al.* 2007) analysis on the human diseasome in the context of a network derived from primary synaptic data within BioNAR framework, which demonstrates how integration of these different Bioconductor packages in BioNAR can enable deeper understanding of the biological system at hand.

The code and datasets for three cases are available from https://github.com/lptolik/BioNAR_paper_supplementary and https://datashare.ed.ac.uk/handle/10283/4793.

## 3 Conclusion

BioNAR provides an analytical pipeline for biological networks, including network import and annotation, estimating scale-freeness and a range of centrality measures, and providing nine different clustering algorithms. It is further extended with methods to estimate network entropy and normalized modularity that can be used to compare networks with different structures. The implementation of bridgeness can be used to estimate the likely importance nodes may have in propagation of signals between different communities. Annotation enrichment features can be used to identify co-occurrence of annotation pairs (typically disease) within a network. Beyond this, the package also allows users to estimate clustering robustness through cross-validation against a selection of randomized network structures.

BioNAR could be extended to improve support for high performance compute clusters for analysis of very large networks; integration with graphical databases, such as Neo4J for more efficient storage, retrieval and querying of networks, and the use of network embeddings, which is a highly active area of research in the AI community. Future releases of BioNAR will also see the inclusion of probabilistic network algorithms based on Bayesian inference to utilize both vertex and edge metadata, including a probabilistic clustering algorithm built from Stochastic Block Models and belief propagation. BioNAR provides researchers with a useful network analysis ‘toolkit’, integrating many network analysis tasks commonly used by the bioinformatics community.

## Supplementary Material

vbad137_Supplementary_Data
