# Supplementary material for: BioNAR: an integrated biological network analysis package in bioconductor
Source: Bioinform Adv. 2023 Sep 29;3(1):vbad137. doi: 10.1093/bioadv/vbad137 (PMC10582516; doi:10.1093/bioadv/vbad137)
Supplement: vbad137_Supplementary_Data [file vbad137_supplementary_data.zip › BioNAR-Supplementary Figure and methods.docx]

##

## Supplementary Figure 1. Power law fit - shown the log-log plot of the CDF of presynaptic DGN network degree distribution (P(k)), versus its degree (k), with the best fitting power-law distribution to the network data highlighted in red.

## Methods

*Clustering.*

Parameters value set in the fc, lec, sg, wt and lourvain algorithms were chosen as to maximise the measure Modularity (Newman and Girvan, 2004); infomap seeks the optimal community structure in the data by maximising the objective function called the Minimum Description Length (Grünwald, et al., 2005; Rissanen, 1978).

The fast-greedy community (fc), Spectral based (lec) and simulated annealing (sg) algorithms have previously been reported to produce a small number of very large communities when applied to real network data, of the order 1000 vertices and 10,000 interactions (McLean, et al., 2016). For the fc algorithm this is a result of the known unbalancing nature of the merging steps (Danon, et al., 2006; Wakita and Tsurumi, 2007), leading to a few large communities growing fast, by merging in many smaller communities. For the lec algorithm this is a result of not including the optional 'fine-tuning' step being applied after each split. Clustering results of the sg algorithm was found to be sensitive to the parameter 'gamma'. The optimal setting for the sg algorithm, to maximise the Modularity, is with gamma is set equal to 1. Increasing gamma (we tested values of gamma = 1,2 and 5) relaxes this condition lowering the Modularity value, but can also reveal the substructure in the optimal clustering results, and help lead to improved functional enrichment results (McLean, et al., 2016). The wt algorithm is known to generate skewed distributions; one or two large communities with many singlet (or two vertex) communities. The skewed distribution is a known cause of the hierarchical approach to clustering and the distribution of the hub (highest degree) vertices inside the network (Akama, et al., 2008; Palla, et al., 2005).

#### Identifying the “influential” proteins

To measure influential genes using the topology of the network, we made use of the semi-local centrality measure $Cl\left( v \right)$of a vertex $v$ (Chen, et al., 2012).

Semi-local centrality measure takes into consideration both a vertex’s degree, its nearest, and next to nearest neighbours:

$$Q\left( u \right)=\sum_{w\in\Gamma\left( u \right)} N\left( w \right)$$

$Cl\left( v \right)=\sum_{u\in\Gamma\left( v \right)} Q\left( u \right)$ (1)

Where $\Gamma\left( u \right)$is the set of nearest neighbours of $u$and $N\left( w \right)$the number of nearest and next to nearest neighbours of vertex $w$. We performed unity-based or feature scaling: X – Xmin/(Xmax-Xmin) to normalise the semi-local centrality to lie in the range [0,1]~~.~~

Semi-local centrality differs from degree centrality therefore, in making use of more information, allowing us to also measure a vertices' spread' of information locally through the network.

To measure the influence of a gene due to the clustering we make use of Bridgeness $B\left( v \right)$of a vertex $v$ (Nepusz, et al., 2008):

$B\left( v \right)=1-\sqrt{\frac{c}{c-1}{\sum_{j=1}^{c} \left( u_{jv}-\frac{1}{c} \right)}^{2}}$ (2)

Where in (2) $u_{v}$is the community membership vector for vertex $v$, that is the probability vertex $v$belongs to a given community:$u_{v}=\left[ u_{1v},u_{2v},...,u_{cv} \right]$where $\sum_{v,j} u_{jv}=1$, and $c$the number of communities detected by the algorithm. For the concrete clustering algorithms given in Tables 5-7, we made use of the consensus clustering and vertex degree to calculate the vertex’s community membership: the approach is similar in spirit to the use of with-in module degree and the participation coefficient in classify the biological importance of proteins in Metabolic networks (Guimerà and Nunes Amaral, 2005).

When plotting Bridgeness (BR) against the semi-local centrality (SLC), the proteins can be categorised by their ability to influence the network “globally and locally”:

- Bridging proteins that are likely to have a 'global' rather than 'local' influence in the network (also been called bottle-neck bridges (Najafi, et al., 2016), connector or kinless hubs (Guimerà and Nunes Amaral, 2005) lie in the range $0\leq Cl\left( v \right)<0.5 and 0.5 \leq B\left( v \right)\leq1$(Region 1).
- Bridging proteins likely to have an influential 'globally' and 'locally' in the network, lie in the range $0.5<Cl\left( v \right)\leq1$and $0.5 \leq B\left( v \right)\leq1$(Region 2).
- Proteins whose impact is mostly 'locally', primarily within one or two communities (local or party hubs (Nepusz, et al., 2008), lie in the range $0.5 \leq Cl\left( v \right)\leq1$ and $and 0 \leq B\left( v \right)\leq0.5$(Region 4).
- Due to disassortative mixing, i.e. a preference for high-degree genes to attach to low-degree genes found in our networks, we found, as expected that most proteins lie in the range,$0\leq Cl\left( v \right)\leq0.5$and $0.1\leq B\left( v \right)\leq0.5$(Region 3), implying most proteins have relatively low influence 'locally' and/or 'globally' in the network. Those proteins with B < 0.1, are what we define as non-Bridging proteins, and contribute as a component to the complex they are found in.

#### Disease network localisation.

We investigated the overlap and separation of each disease-disease pair by measuring the mean shortest distance for each disease ($\langle d\rangle$), using the shortest distance between each GDA to its next nearest GDA neighbour (Menche, et al., 2015). The overlap, or separation, of each disease-disease pair in the pre- post-synaptic PPI networks, could then be quantified using:

$s_{AB}\equiv\langle d_{AB}\rangle-\frac{\langle d_{AA}\rangle+\langle d_{BB}\rangle}{2}$ - (6)

Where $\langle d_{AA}\rangle$and $\langle d_{BB}\rangle$quantify the mean shortest network distance between genes associated with disease A (or B) , and $\langle d_{AB}\rangle$the mean shortest distance between diseases. $s_{AB}$Is bound by the diameter of the network, i.e. $d_{max}\leq s_{AB}\leq d_{max}$, where $d_{max}$is 8, 7, 8 for the presynaptic, PSP and PSP reduced PPI networks respectively. The magnitude of $s_{AB}$depends on the number of GDSs associated with each disease. Large positive values imply two well separated diseases, while large negative values indicate large (number of GDAs) diseases with a big overlap, often implying one disease is the variant or precursor to the other. Each disease-disease network separation pair ($s_{AB}$) was compared against a full randomised model: drawing the same number of GDAs (from the set of all network genes) for each disease at random, before computing its separation $s_{AB}^{rand}$. For each disease-disease pair, we performed 1000 iterations of the full randomised model using the ECDF distributed computing facility.

The difference between the observed and randomised disease pair separations, was quantified using the z-score:

$z-{score}_{AB}=\frac{s_{AB}-\langle s_{AB}^{rand}\rangle}{\sigma\left( s_{AB}^{rand} \right)}$- (7)

Where $\langle s_{AB}^{rand}\rangle$and $\sigma\left( s_{AB}^{rand} \right)$are the mean and standard deviation obtained from the 1000 iterations. Each disease-disease pair separation using the full randomised model, i.e.$s_{AB}^{rand}$, was found to follow a normal distribution. We therefore assessed the significance of each disease-disease pair's separation, from P-values estimated from its z-score calculated in (7):

$P-value\left( X=-\left| Z-{score}_{AB} \right|;\mu=0,\sigma=1 \right)=\left[ 1+erf\left( \frac{x-\mu}{\sigma\sqrt{2}} \right) \right]$- (8)

Where we take the negative of the absolute value of each disease-disease pairs z-score calculated in (7) and make use of R's pnorm function available in the 'stats' package (R version 3.4.2).

The confidence in each disease-disease pairs P-value was tested for by calculating its q-value (Storey and Tibshirani, 2003), and from the Bonferroni correction at the 0.05 (*), 0.01 (**) and 0.001 (***) significant levels. A description on how each disease-disease pairs q-values was estimated is described in the Supplementary Analysis.
